# Intratumoral STING activation causes durable immunogenic tumor eradication in the KP soft tissue sarcoma model

**DOI:** 10.3389/fimmu.2022.1087991

**Published:** 2023-01-09

**Authors:** Kayla L. Marritt, Karys M. Hildebrand, Kurt N. Hildebrand, Arvind K. Singla, Franz J. Zemp, Douglas J. Mahoney, Frank R. Jirik, Michael J. Monument

**Affiliations:** ^1^ Department of Surgery, Cumming School of Medicine, University of Calgary, Calgary, AB, Canada; ^2^ McCaig Bone and Joint Institute, Cumming School of Medicine, University of Calgary, Calgary, AB, Canada; ^3^ Arnie Charbonneau Cancer Research Institute, Cumming School of Medicine, University of Calgary, Calgary, AB, Canada; ^4^ Department of Microbiology, Immunology and Infectious Diseases, Cumming School of Medicine, University of Calgary, Calgary, AB, Canada; ^5^ Alberta Children’s Hospital Research Institute, University of Calgary, Calgary, AB, Canada; ^6^ Department of Biochemistry and Molecular Biology, Cumming School of Medicine, University of Calgary, Calgary, AB, Canada

**Keywords:** cancer immunotherapy, cGAS/STING, undifferentiated pleomorphic sarcoma, KP sarcoma model, soft tissue sarcoma

## Abstract

**Introduction:**

Soft tissue sarcomas (STS) are highly metastatic, connective-tissue lineage solid cancers. Immunologically, sarcomas are frequently characterized by a paucity of tumor infiltrating lymphocytes and an immune suppressive microenvironment. Activation of the STING pathway can induce potent immune-driven anti-tumor responses within immunogenic solid tumors; however, this strategy has not been evaluated in immunologically cold sarcomas. Herein, we assessed the therapeutic response of intratumoral STING activation in an immunologically cold murine model of undifferentiated pleomorphic sarcoma (UPS).

**Materials and Results:**

A single intratumoral injection of the murine STING agonist, DMXAA resulted in durable cure in up to 60% of UPS-bearing mice. In mice with synchronous lung metastases, STING activation within hindlimb tumors resulted in 50% cure in both anatomic sites. Surviving mice all rejected UPS re-challenge in the hindlimb and lung. Therapeutic efficacy of STING was inhibited by lymphocyte deficiency but unaffected by macrophage deficiency. Immune phenotyping demonstrated enrichment of lymphocytic responses in tumors at multiple timepoints following treatment. Immune checkpoint blockade enhanced survival following STING activation.

**Discussion:**

These data suggest intratumoral activation of the STING pathway elicits local and systemic anti-tumor immune responses in a lymphocyte poor sarcoma model and deserves further evaluation as an adjunctive local and systemic treatment for sarcomas.

## Introduction

Soft tissue sarcomas (STS) are rare malignancies derived from mesenchymal lineage tissues such as muscle, adipose, fibrous tissue, vessels, and skin (1). Sarcomas are rare, representing <1% of all cancer diagnoses, yet disproportionately account for 15-20% of solid cancers in children, adolescents, and young adults (2–4). There are over 50 unique histologic subtypes of STS (1), with undifferentiated pleomorphic sarcoma (UPS) being the most common subtype in adults (4). High-grade soft tissue sarcomas are considered a high-fatality disease characterized by frequent metastases, resistance to systemic therapies, and a five-year survival rate of under 60% (1, 5). Unresectable metastatic disease is rapidly fatal (1, 6–8) and there is a pressing need for new systemic therapies for STS patients (9).

Immunotherapies are revolutionizing cancer care (10–13), yet unfortunately, sarcoma remains recalcitrant to multiple clinically approved immune-based therapies (14–18). Relative to other solid cancers, most sarcomas are deficient in tumor infiltrating lymphocytes (TILs) (19–21), which, like other solid cancers, predicts poor therapeutic responses to immune checkpoint inhibition (ICI) (22). The immunosuppressive landscape of STS is multifactorial and can be attributed to a combination of low tumor mutational burden, dense infiltration of immune suppressive macrophages (“M2-like” macrophages), and the expression of immune suppressive connective tissue cytokines and growth factors within mesenchymal-derived sarcomas (23–25).

The stimulator of interferon genes (STING) receptor is a highly conserved intracellular protein involved in the dsDNA sensing apparatus of eukaryotic cells and is responsible for Type I IFN and cytokine production in response to cytosolic DNA derived from pathogens and corrupt host cells (26, 27). The STING pathway provides a critical link between the innate and adaptive compartments of the immune system and is a vital component of cancer immunity (19, 21, 28). When STING is activated, the potent liberation of Type I IFNs and other inflammatory mediators results in tumor necrosis (19, 28), activation of antigen presenting cells (APCs) (25, 28), enhanced cross-priming of CD8^+^ lymphocytes and recruitment of anti-tumor lymphocytes into the tumor immune microenvironment (TIME) (19, 21, 28). In pre-clinical models of classically inflamed solid tumors, intratumoral (i.t.) small molecule STING agonists can induce dramatic local tumor regression and systemic immunity against distant disease and this strategy has now entered early phase clinical trials.

STING immunotherapy has not been evaluated in immunogenically cold models of STS. As poorly inflamed sarcomas are recalcitrant to immune-based therapies such as immune checkpoint inhibitors (14, 17–19, 21), we hypothesized that i.t. STING therapy would be an effective strategy to dismantle the immune suppressive sarcoma microenvironment and sensitize murine STSs to ICI. Herein, we evaluated the therapeutic anti-tumor effects of STING activation in a lymphocyte poor murine model of UPS that is resistant to ICI (29–31). We demonstrate that that a single i.t. dose of a small molecule STING agonist resulted in rapid immune-mediated tumor clearance locally and systemically and therapeutic synergy with immune checkpoint blockade.

## Materials and methods

### Mice

All *in vivo* murine studies were performed animal use protocols approved by the University of Calgary Health Sciences Animal Care Committee (#AC19-0072). Mice were housed in a biohazard level 2 containment facility in individual cages (Techniplast) equipped with HEPA filters and filtered air. The mouse housing room was maintained at 22 +/- 1°C, 30-35% humidity, and was on a 12-hour light/dark cycle. The mice were allowed standard food and water *ad libitum*. All *in vivo* murine experiments were performed in 6–8-week-old male and female mice. All mice were purchased from Jackson Laboratories and then bred in house. Rag2 KO mice (B6(Cg)-Rag2^tm1.1Cgn^/J Rag2 knockout mice; stock #008449) are deficient in mature T-cells and B-cells (32). CCR2 KO mice (B6.129S4-*Ccr*2^tm1Ifc^/J CCR2 knockout mice; stock #027619) show a monocyte recruitment deficiency to sites of inflammation and were used to test tumor macrophage deficiency (33).

### Tumor model

The development of the syngeneic KP UPS cell line used in these experiments is described and characterized by Hildebrand et al. (2021) (29), and also previously by DuPage et al. (2012) (31) and Kirsch et al. (2007) (30). Briefly, spontaneous UPS tumors were generated in conditional *Trp53^fl/fl^* and *Kras^G12D/+^* mice *via* lenti-Cre (University of Iowa Viral Vector Core; FIVCMVCre VSVG) mediated *Trp53* deficiency and activation of the Kras^G12D^ oncogene subperiosteally in the hindlimb of female C57Bl/6 mice which results in establishment of primary UPS tumors exclusively in the proximal tibia of the hindlimb. Following a latency of 8-10 weeks, hindlimb tumors were harvested and cultured *in vitro* for 6-8 weeks for cell line development. Only cell line derived tumors were used as the model for this project. Cultured UPS tumor cells were engineered to express mCherry and firefly luciferase *via* transduction with pLV430G oFL T2A mCherry vector. This cell line is referred to as “TAO1+”. Aliquots of UPS cell not transduced with mCherry and luciferase are referred to as “TAO1-”. All UPS tumors evaluated in *in vivo* experiments reported in this study utilized engrafted TAO1+ UPS tumors in which UPS cells were resuspended in serum free RPMI-1640 media and injected intramuscularly into the right hindlimb. The quantity of UPS cells injected were as follows: 100,000 for primary injection, 10,000 for contralateral limb injection, and 100,000 for tail vein injection.

### Tumor volume assessment and bioluminescent imaging

Tumors were monitored by caliper measurements and bioluminescent imaging (BLI). For BLI, mice were injected with D-luciferin (Goldbio Technology; cat. #LUCK-1G) intraperitoneally and imaged using a Xenogen IVIS Lumina system (Caliper Life Sciences, Hopkinton, MA, USA) ten minutes following injection. Living Image Software (PerkinElmer) to collect and analyze the BLI images. The image exposure was set to “Auto.”

Caliper measurements were used to measure tumor length, width, and depth. Length is defined as proximal to distal, width is defined as lateral to medial, and depth is defined as anterior to posterior measurements. Tumor volumes were calculated with the formula (L+X)*L*X*0.2618, where L is the length of the tumor and X is (width of tumor + depth of tumor)/2 (29, 34). The humane endpoint for any mouse experiment was defined as a leg tumor exceeding 15 mm in the length, width, or depth dimensions. For the tail vein injection experiments and any mice with lung tumors, the humane endpoint was defined as any rapid deterioration of overall health including rapid weight loss, loss of grooming, hunched posture, and lethargic behavior. Experimental endpoint for any murine long-term survival experiment was defined as three months after primary cell line injection, one month after contralateral limb re-challenge, and two months after tail vein re-challenge. All mice alive beyond these experimental timelines are regarded as “survivors.” We have not observed any evidence of UPS relapse after these experimental endpoints.

### 5’6-dimethylxanthenone-4-acetic acid experiments

In this study, DMXAA was used to investigate STING immunotherapy in murine UPS tumors. In all experimental groups, 100,000 UPS cells were injected into the right hindlimb muscle of C57Bl/6 mice. Intra-tumoral (i.t.) injection(s) of DMXAA (Sigma; cat. #D5817-25MG) or sodium bicarbonate (Gibco; cat. #25080094) were administered when UPS tumors reached ~100 mm^3^ (7 days after cell line engraftment). The experimental groups were: (i) one DMXAA (18 mg/kg) injection (n=10), (ii) one DMXAA (25 mg/kg) injection (n=14), (iii) two DMXAA (18 mg/kg) injections (n=10), (iv) three DMXAA (18 mg/kg) injections (n=10), and (v) sodium bicarbonate vehicle controls (n=9). For (i), (ii), and (v) the treatment was delivered 7 days post UPS injection. For experiment (iii) DMXAA was administered 7- and 14-days post UPS injection. For experiment (iv) DMXAA was administered 7-, 11-, and 14-days post UPS injection. An additional cohort was utilized in which 100,000 UPS cells were injected into the tail vein for lung engraftment on day 0, followed by concurrent leg tumor engraftment of 100,000 UPS cells on day 7. On day 14, 18 mg/kg of DMXAA was administered i.t. in the hindlimb. Single and double DMXAA doses were chosen based on previous studies (24, 28, 35). The triple DMXAA dosing was modified from this same study. For the Rag2 and CCR2 KO mice experiments, 100,000 UPS cells were injected into the right hindlimb muscle. DMXAA (18 mg/kg) or sodium bicarbonate vehicle control were injected i.t. when tumors reached ~100 mm^3^ (7 days after cell line engraftment).

### *In vivo* re-challenge experiments

Mice from the cohort that were engrafted with 100,000 UPS cells in the right hindlimb, subsequently received an i.t. dose of DMXAA (18 mg/kg) and survived were re-challenged with UPS cells. Survival was characterized as mice that are tumor free with no evidence of tumor after three months. For the re-challenge experiments in the primary site, 10,000 UPS cells were injected into the muscle of the contralateral hind limb of “survivors” and naïve C57Bl/6 mice. For the tail vein re-challenge experiments, 100,000 UPS cells administered through a tail vein injection in “survivors” and naïve C57Bl/6 mice. Tail vein injections of murine UPS cells into C57Bl/6 mice had been previously determined by our laboratory to result in UPS tumors exclusively in the lung within 3-4 weeks using this model. Weekly BLI and overall mouse health were used to assess tumor growth.

### Immune checkpoint inhibitor therapy

Mice bearing syngeneic UPS hindlimb tumors were treated with a mouse anti-CTLA4 monoclonal antibody (BioXcell, CD152, clone 9D9, 250 μg) or a mouse anti-PD1 monoclonal antibody (BioXcell, CD279, clone RMP1-14, 250 μg) intraperitoneally, on days 7, 10, and 13 following UPS injection. For anti-PD-1 + anti-CTLA4 dual therapy, UPS-bearing mice were treated with mouse anti-CTLA4 (BioXcell, CD152, clone 9D9, 250 μg) and mouse anti-PD1 (BioXcell, CD279, clone RMP1-14, 250 μg) intraperitoneally, on days 9, 11, 15, 18, 22, 25, 29, and 32 following UPS injection. For DMXAA + anti-PD-1 + anti-CTLA4 combination therapy, UPS-bearing mice were treated with i.t. DMXAA (18 mg/kg) on day 7 and mouse anti-CTLA4 (BioXcell, CD152, clone 9D9, 250 μg) and mouse anti-PD1 (BioXcell, CD279, clone RMP1-14, 250 μg) intraperitoneally, on days 9, 11, 15, 18, 22, 25, 29, and 32 following UPS injection.

### Flow cytometry

100,000 UPS cells were engrafted into the hind limb muscle of C57Bl/6 mice and 7 days later when the tumors reached a tumor volume of ~100 mm^3^, i.t. injections of DMXAA (18 mg/kg) or sodium bicarbonate were given. Tumors were processed for flow cytometry 3- and 7-days post DMXAA or sodium bicarbonate treatment. UPS tumors were excised and homogenized using a gentleMACS Dissociator (Miltenyi). Tumors were digested with RPMI-1640 media (Gibco; cat. #22400089) containing 0.5 mg/mL DNAse I (Roche Diagnostics; cat. #10104159001), 20 mg/mL Collagenase II (Gibco; cat. #17101-015), and 0.5 mL/10 mL fetal bovine serum (Gibco; cat. #12483-020). Tumors were then strained with a 70 μm strainer (Falcon™; cat. #08-771-2), treated with RBC lysis buffer (Biolegend; cat. #420301), and washed with 40% Percoll™ (cat. #17-0891-02).

Single cell suspensions were stained with LIVE/DEAD Zombie Aqua (cat. #423102) before antibody staining for 15-30 minutes. Antibody staining was completed using the following fluorophore-conjugated antibodies: CD3ϵ (cat. #155609), CD4 (cat. #100512), CD8α (cat. #100733), CD45 (cat. #103154), CD11b (cat. #101207), Ly6C (cat. #128005), and Ly6G (cat. #127615). Data was acquired using a FACSCanto II (BD Biosciences) with FACSDiva software (BD Biosciences). The data was analyzed with FlowJo (TreeStar). T-cells were defined as CD3ϵ+/CD4+ (CD4 T-cells) and CD3ϵ+/CD8+ (CD8 T-cells). Monocytes were defined as CD45+/CD11b+/Ly6C+/Ly6G-, neutrophils as CD45+/CD11b+/Ly6C+/Ly6G+ and macrophages as CD45+/CD11b+/Ly6C-/Ly6G-. Controls included a dead cell sample, achieved by heating the tumor cells to 80°C for 15 minutes, unstained tumor cells, and single colour controls. Single colour controls were made using compensation beads (Invitrogen) (cat. #501129040).

### mRNA quantification and analysis

NanoString^®^ technology was used to compare the mRNA expression levels of ~750 genes in the following four treatment groups: control UPS tumors (n=4), UPS tumors 24 hours post DMXAA treatment (18 mg/kg; n=4), and UPS tumors 72 hours post DMXAA treatment (18 mg/kg; n=7). Total RNA was extracted from TAO1+ UPS tumors using standard protocols. 100 μg of unamplified total RNA input was used for codeset hybridization using the mouse-specific nCounter^®^ PanCancer Immune Profiling panel (NanoString^®^ Technologies, Seattle, WA) (36). Codeset/RNA complexes were immobilized on nCounter^®^ cartridges for data collection. nSolver Analysis Software 4.0 and Advanced Analysis were used for analysis and figure generation.

### Histopathology

UPS tumors were fixed in 10% neutral buffered formalin (Research Products International Corp) for 24 hours and embedded into paraffin using a tissue processor (Leica). The tissues were sectioned to 5 microns (Leica RM2255) and stained with hematoxylin and eosin (H&E) following the same protocol as Foothills Medical Centre Calgary Laboratory Services.

### Statistical analysis

For survival plots, the log-rank Mantel-Cox test was used. For categorical variables, a two-way ANOVA with Bonferroni’s multiple comparisons test was used. The development of all graphs as well as statistical analysis was performed using GraphPad Prism version 8.2.1.

## Results

### Intratumoral STING activation induces durable survival in UPS-bearing mice

**Figure 1:**
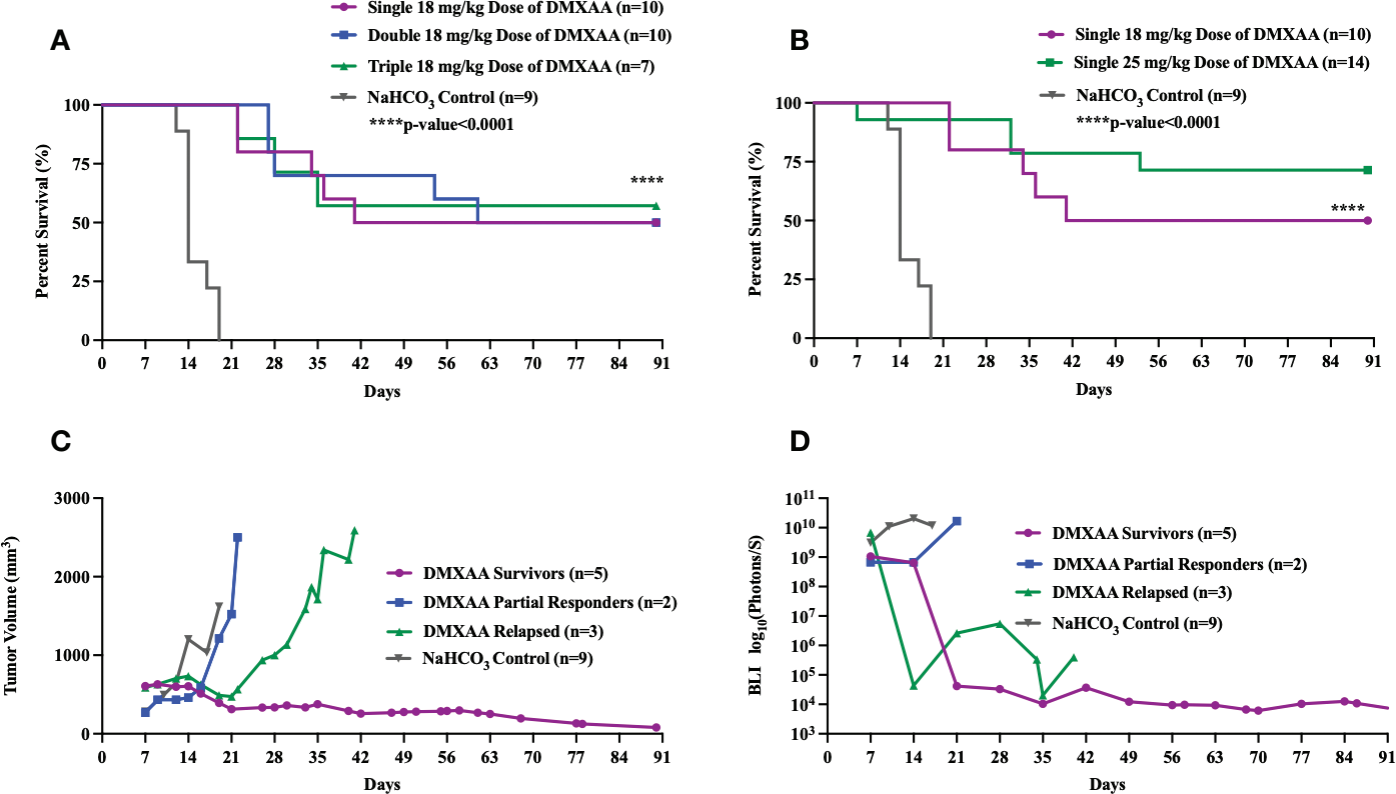
Intratumoral STING activation results in long-term survival in UPS-bearing C57Bl/6 mice. **(A-D)** 100,000 UPS + mCherry and luciferase cells were injected intramuscularly on day 0. **(A)** DMXAA (18 mg/kg) was injected i.t. according to varying dose schedules: single dose = injected on day 7, double dose = injected on days 7 and 14, and triple dose = injected on days 7, 11, and 14. **(B)** DMXAA (18 mg/kg or 25 mg/kg) or NaHCO_3_ was injected i.t. on day 7. Mean tumor volume **(C)** and mean BLI **(D)** of UPS-bearing C57Bl/6 mice treated with DMXAA. **(C, D)** 18 mg/kg DMXAA or NaHCO_3_ was injected i.t. on day 7.

DMXAA is an established murine-specific STING agonist with known dosing parameters and minimal toxicities below 30 mg/kg (28, 37). We first sought to determine if different dosing schedules of DMXAA would result in therapeutic anti-tumor effects. Single, double (3 days apart) or triple (every three days) i.t. doses of DMXAA resulted in complete tumor eradication beyond 3 months in 50-60% of mice (**Figure 1A**). I.t. dosing of DMXAA was chosen over intra-peritoneal administration to maximize local induction of i.t. immune responses. Additionally, there are reports that i.t. DMXAA is more effective at activating STING responsiveness in tumors than i.p. administration (28). We observed greatest tumor eradication in the triple dosed cohort but did observe overlying skin necrosis in over 50% of these mice. There we no observed toxicities in the 18 mg/kg group. Using a 25 mg/kg single i.t. dose we observed increased complete tumor eradication (70%) compared to the 18mg/kg dose (50%), although one mouse died from presumed treatment toxicity within 24hrs of injection (**Figure 1B**).

All DMXAA treated tumors showed immediate tumor volume and BLI reductions compared to control. A more detailed examination of tumor volumes and tumor BLI data in the single 18 mg/kg treated mice shows three distinct patterns of response to DMXAA treatment: long-term survivors, partial responders, and late relapse (**Figures 1C, D**). In the partial responder group, the mean tumor volumes steadily increased after a transient reduction (**Figures 1C, D**). In the relapsed group, mean tumor volume and BLI signal steadily decreased, and the tumors were no longer palpable, however around day 28, tumors became palpable again with associated increased BLI signal (**Figures 1C, D**).

### UPS re-challenge is rejected in STING-treated surviving mice

**Figure 2:**
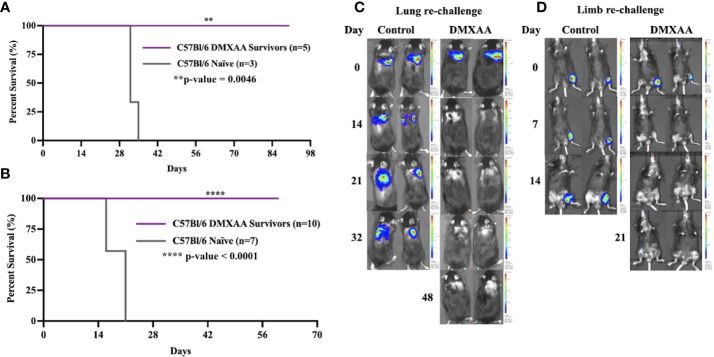
Intratumoral STING activation provides protective immunity against UPS re-challenge. **(A**, **C)** C57Bl/6 DMXAA survivors and naïve C57Bl/6 mice were given 100,000 UPS TAO1+ mCherry and luciferase cells injected *via* tail vein on day 0. **(B**, **D)** “Survivors” and naïve C57Bl/6 mice were re-challenged with 10,000 UPS TAO1+ cells injected intramuscularly into the contralateral limb on day 0. BLI images of tail vein re-challenge **(C)** and contralateral limb re-challenge **(D)** in naïve C57Bl/6 mice (control) and DMXAA survivors (DMXAA). **p-value=0.0046. ****p-value<0.0001.

We next sought to determine if successful clearance of UPS tumors following STING therapy would result in systemic protection against UPS recurrence. To mimic the clinical scenario of sarcoma recurrence in the extremity (local) or lung (metastatic), we performed UPS re-challenge experiments on previous UPS-bearing mice that completed eradicated their tumors after STING therapy. “Survivor” mice were re-challenged with UPS cells in either the contralateral limb or lung resulted and 100% of these mice rejected the UPS re-challenge as defined by no BLI signal or palpable tumor for up to 60 days of observation (**Figure 2**). All control mice in these experiments developed hindlimb and lung tumors that rapidly progressed to humane endpoint (**Figure 2**). There were no differences in UPS tumor clearance between males and females (**Figure S1**).

### STING activation in extremity UPS tumors results in systemic clearance of limb tumors and synchronous lung lesions

**Figure 3:**
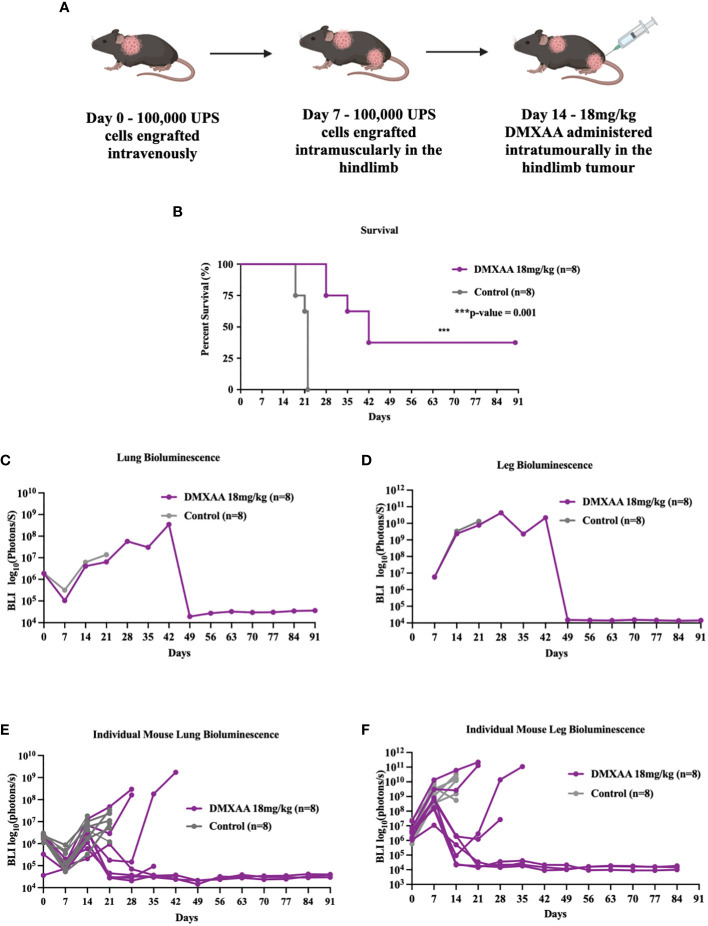
STING treatment of extremity UPS results in systemic eradication of synchronous lung metastases. **(A)** A schematic outlining the establishment of UPS tumors in the lung on day 0. 100,000 UPS TAO1+ mCherry and luciferase cells were injected in the tail vein, subsequently followed by leg tumor engraftment on day 7, and treatment of the hindlimb tumors with 18 mg/kg of DMXAA or vehicle control sodium bicarbonate i.t. on day 14. **(B)** Kaplan Meier plot comparing the survival of DMXAA, and vehicle control treated mice. **(C, D)** BLI intensity of leg and lung tumors in DMXAA and vehicle control groups. **(E, F)** BLI intensity of leg and lung tumors in DMXAA and vehicle control tumors individually.

The lung is the most common site of metastases in STS. To evaluate if STING treatment of extremity UPS tumors could also induce therapeutic responses in sites of distant disease, we tested STING activation in a model of synchronous hindlimb and lung tumors. Naïve mice were engrafted with UPS cells in the lung *via* tail vein injections (Day 0), followed by UPS engraftment in the right hindlimb (Day 7), and then given DMXAA (Day 14; 18 mg/kg) i.t. (**Figure 3A**). All mice developed engrafted UPS tumors in the lung and hindlimb as detected by BLI imaging. Mice bearing simultaneous hindlimb and lung UPS tumors that received i.t. STING therapy all survived longer than control mice, with 30% of STING treated mice completely eradicating UPS tumors in both anatomic sites (**Figure 3B**). By day 49, all surviving mice had complete and durable tumor remission in both sites (**Figures 3C, D**). Examining individual BLI data, 50% of mice that did not survive STING therapy developed severe tumor burden in the lung, and similar to isolated hindlimb DMXAA experiments, some mice transiently cleared the lung tumors only to relapse around 3 weeks post-therapy (**Figure 3E**).

### Intratumoral STING activation results in tumor necrosis, lymphocyte infiltration, and upregulation cytotoxic adaptive immune pathways

**Figure 4:**
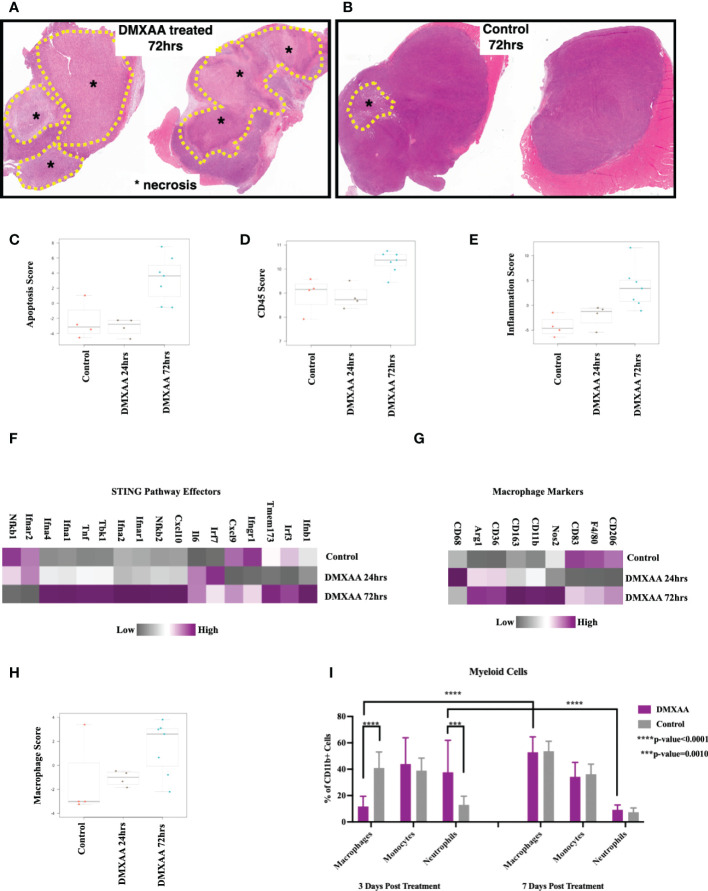
Intratumoral STING activation results in necrosis and upregulation of apoptotic and myeloid markers. **(A, B)** Low magnification microscopy of hematoxylin and eosin (H&E) stained tumor mid-sections shows substantial tumor necrosis 72hrs post STING therapy in UPS tumors. Nanostring nSolver^®^ analyses of immune mRNA transcripts demonstrating increased apoptosis **(C)**, leukocyte infiltration **(D)**, and tumor inflammation **(E)** within 72hrs of STING therapy. nSolver^®^ generated heatmaps show increased mRNA expression profiles of common STING pathway and effectors markers **(F)**, macrophage markers **(G)**, and macrophage functional scores **(H)** 72hrs after STING therapy. **(I)** FACS analyses of tumor cell suspensions for myeloid cells (CD45+, CD11b+), which includes macrophages (Ly6G-, Ly6C-), monocytes (Ly6G-, Ly6C+), and neutrophils (Ly6G+, Ly6C+).

To elucidate the changes within the UPS TIME following STING therapy, ex-vivo analyses of DMXAA treated UPS tumors were evaluated at multiple time points after treatment. Mid-tumor H&E sections showed >50% necrosis in all DMXAA treated tumors at 72hrs, with minimal spontaneous necrosis in control tumors (**Figures 4A, B**). Transcriptomic analyses also demonstrated higher apoptotic pathway scores was at 72hrs post STING treatment compared to control (**Figure 4C**).

Using FACS and Nanostring^®^ transcriptome analyses, we sought to evaluate changes in immune populations within UPS tumors following STING treatment at various timepoints. Overall leukocyte infiltration and general inflammation scores were increased within 72hrs of STING treatment (**Figures 4D, E**). Additionally, there was elevated mRNA expression of downstream markers associated with the STING pathway or effectors of STING activation (**Figure 4F**), such as *Tbk1*, *Irf3*, as well as interferons alpha-1, 2, and 4 (*Ifna1, 2, and 4*), beta-1 (*Ifnb1*), and gamma receptor (*Ifngr*), thus further confirming evidence of persistent upregulation of STING pathway and effectors up to 72hrs following DMXAA treatment.

Assessing the myeloid immune compartment, gene expression levels of most macrophage markers were decreased at early time points post STING therapy but rebounded and were elevated relative to control by 72hrs (**Figure 4G**). Mean macrophage function scores were also increased 72hrs post STING treatment compared to control UPS tumors (**Figure 4H**). Using FACS we observed a rapid increase in neutrophils at early timepoints following STING treatment, which like the mRNA analyses, was associated with a reciprocal reduction in macrophages as well. This trend, however, was reversed by 7 days, where macrophage numbers steadily increased and neutrophil numbers declined (**Figure 4I**).

**Figure 5:**
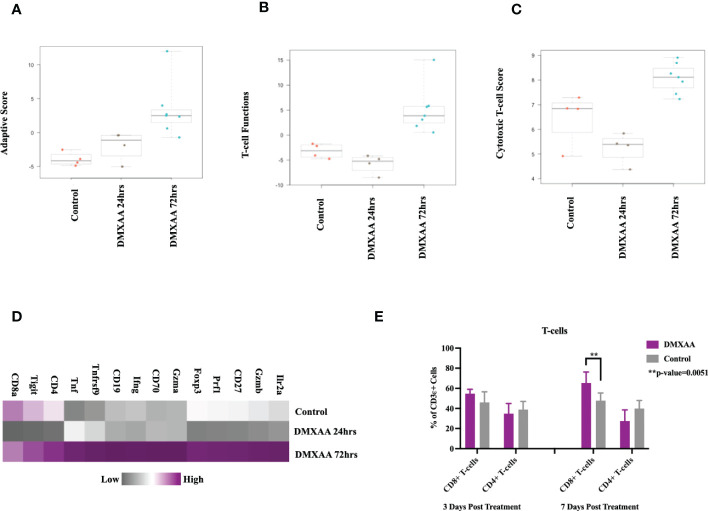
Intratumoral STING activation results in upregulation of lymphocytic markers and infiltration of cytotoxic T-lymphocytes. nSolver^®^ advanced analysis of STING treated of UPS tumors demonstrates increased **(A)** adaptive immune pathway scores, **(B)** T-cell function scores, and **(C)** cytotoxic T-lymphocyte scores. Heat maps illustrate the **(D)** increased adaptive and cytotoxic mRNA expression profiles observed in UPS tumors following STING treatment. **(E)** FACS analyses of tumor cell suspensions assessing CD8 T cells (CD3ϵ+, CD8+) and CD4 T cells (CD3ϵ+, CD4+).

Examining the adaptive immune compartment, STING treated tumors demonstrated an elevation in adaptive immune scoring of mRNA expression profiles 72hrs after treatment (**Figure 5A**). T cell function scores and cytotoxic scores of mRNA analytes were also elevated in the 72hrs post DMXAA treatment group (**Figures 5B, C**). Direct mRNA expression levels of common lymphocyte markers were most upregulated in the 72hrs post DMXAA treatment in UPS tumors when compared to the control UPS tumors and 24hrs post DMXAA treatment (**Figure 5D**). There was a higher expression of *Cd3ϵ*, *Cd4*, and *Cd8* in control and tumors 72hrs after DMXAA treatment compared to 24hrs after treatment. However, there was an elevated expression of cytotoxic markers (Granzymes A and B; *Gzma* and *Gzmb*, Figure D) in tumors 72hrs after DMXAA treatment. Using FACS, compared to control, increased ratios of CD8+ T-cells were also observed in the STING treated UPS tumors seven days after treatment, while the quantity of CD4+ T-cells remained stable across all time points (**Figure 5E**).

Collectively, these investigations of the UPS TIME demonstrate that i.t. STING activation results in tumor necrosis, liberation of STING effector chemokines and cytokines, early neutrophil influx, followed by increases in adaptive immunity gene expression and CD8+ lymphocyte infiltration.

### Lymphocyte deficiency, but not macrophage deficiency, attenuates anti-tumor benefits of intratumoral STING immunotherapy

**Figure 6:**
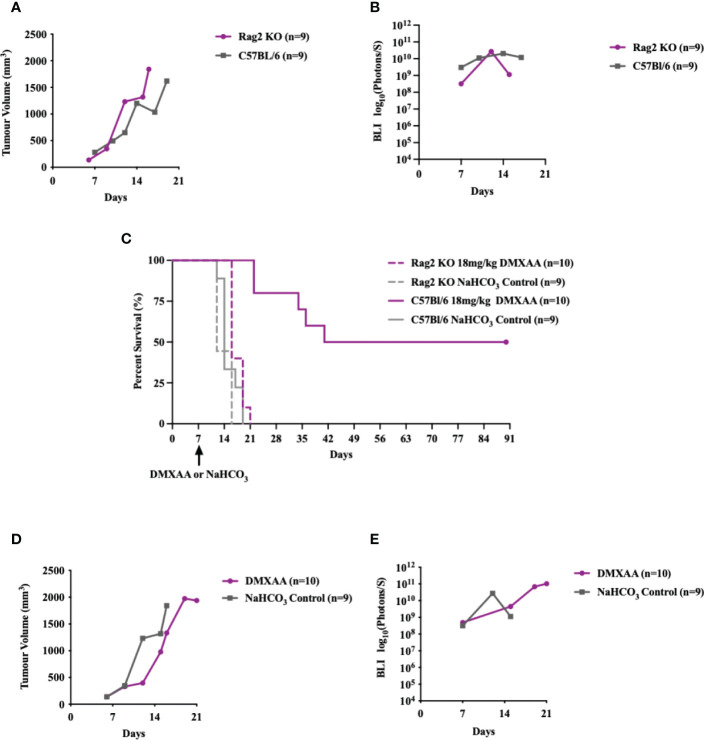
Intratumoral STING activation and subsequent anti-UPS tumor effects are mediated by adaptive immune responses. **(A-E)** 100,000 UPS + mCherry and luciferase cells were injected intramuscularly on day 0. **(A)** Mean tumor volume and **(B)**–mean BLI ROI of UPS growth in Rag2 KO mice (purple) and C57Bl/6 mice (grey). **(C)** Survival of untreated Rag2 KO (solid purple) and C57Bl/6 mice (solid grey), as well as Rag2 KO (dashed purple) and C57Bl/6 mice (dashed grey) treated i.t. with DMXAA (18 mg/kg) on day 7. **(D)** Mean tumor volume and **(E)** mean BLI ROI of Rag2 KO mice (purple) and C57Bl/6 mice (grey) treated i.t. with DMXAA (18 mg/kg) on day 7.

To determine if STING-mediated tumor clearance is dependent on an adaptive immune response, we tested DMXAA treatment in Rag2 Knockout (KO) mice (**Figure 6**). UPS engraftment, growth kinetics and time to humane endpoint were unaffected by lymphocyte deficiency (**Figures 6A, B**). There was also no significant difference between the overall survival time (p-value = 0.1728) of UPS bearing Rag2 KO mice and C57Bl/6 mice (**Figure 6C**). These findings suggest negligible engagement of the adaptive immune compartment in the progression of tumor growth or engraftment in this UPS model. The anti-tumor effects of STING therapy, however, were lost when UPS engrafted Rag 2 KO mice were treated with intra-tumoral DMXAA (**Figures 6C-E**). A marked decrease in UPS tumor volume was observed in DMXAA treated Rag2 KO mice (days 7-14; **Figure 6C**), with tumor volumes sharply rebounding afterwards. These observations would suggest early tumor clearance following STING therapy *via* lymphocyte independent mechanisms, although UPS tumors could not be cleared beyond 14 days without an intact lymphocyte compartment.

**Figure 7:**
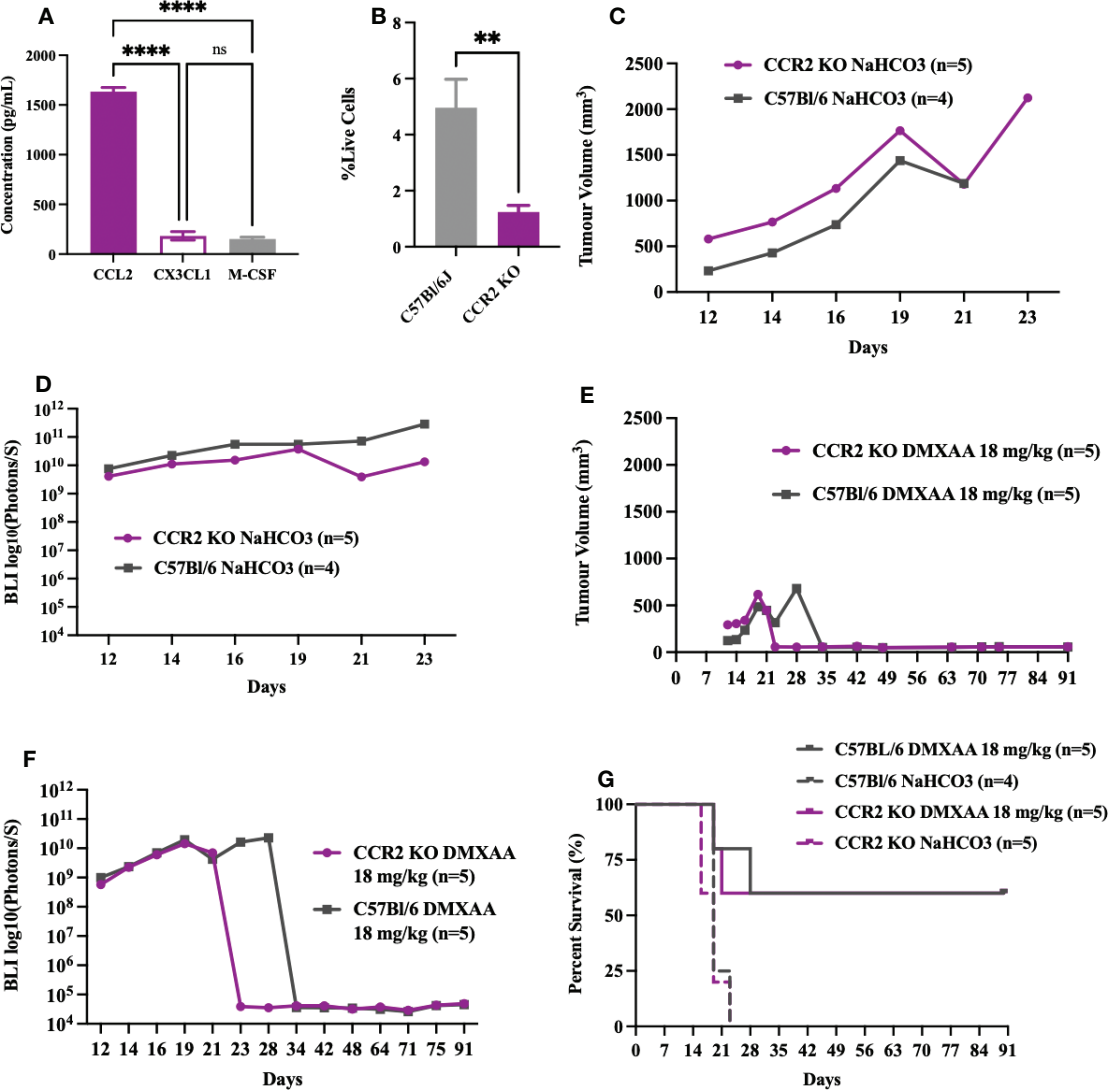
Impairing monocyte recruitment with CCR2 deficiency showed similar UPS response to intratumoral STING activation. **(A)** Concentration of monocyte chemoattractants (CCL2, CX3CL1, M-CSF) in the supernatant of UPS cell culture. **(B-G)** 100,000 UPS + mCherry and luciferase cells were injected intramuscularly on day 0. **(B)** UPS tumor macrophages (CD45+, CD11b+, F4/80+) in CCR2 KO mice are reduced by 75% compared to control C57Bl/6 mice 9-days after UPS engraftment. **(C)** Mean tumor volume and **(D)** mean BLI ROI of UPS tumor growth curves in CCR2 KO mice and C57Bl/6 mice. **(E)** Tumor volume and **(F)** mean BLI ROI of UPS-bearing CCR2 KO or C57Bl/6 mice treated with 18 mg/kg DMXAA i.t. on day 7. **(G)** Longitudinal *in vivo* survival of UPS bearing mice following STING therapy showing similar overall survival in CCR2 KO and control C57Bl/6 mice. ns, non-significant. **p-value=0.043. ****p-value<0.0001.

As STS are highly enriched in macrophages and given that macrophages are highly responsive to STING agonists (37–39), we sought to determine if reductions in UPS macrophages would mitigate the early or innate immune response to DMXAA. The CCR2/CCL2 is a known recruitment axis for tumor associated macrophages and highly expressed by TAO1 cells in culture (**Figure 7A**) we utilized a CCR2 KO model, which leads to deficiencies in monocyte recruitment into tumors (33) and has been shown reduced tumor macrophages in previous work (40). Engrafted UPS tumors in CCR2 KO mice showed 75% reduction of macrophages in UPS tumors (**Figure 7B**), but no differences in tumor growth kinetics and time to humane endpoints (**Figures 7C, D**). Following i.t. DMXAA, both control and CCR2 KO mice showed reduction in UPS tumor volumes (**Figures 7E**), tumor bioluminescence (**Figure 7F**), and tumor free survival 90-days post-UPS engraftment (**Figure 7G**). However, UPS tumors in the CCR2 KO group demonstrated quicker tumor volume and BLI response to treatment (**Figures 7E, F**). These results would suggest that tumor macrophage reductions *via* the CCR2/CCL2 axis did not impair responsiveness to STING agonist therapy and may have promoted a more rapid early/innate response.

### STING therapy is synergistic with immune checkpoint blockade in murine UPS

**Figure 8:**
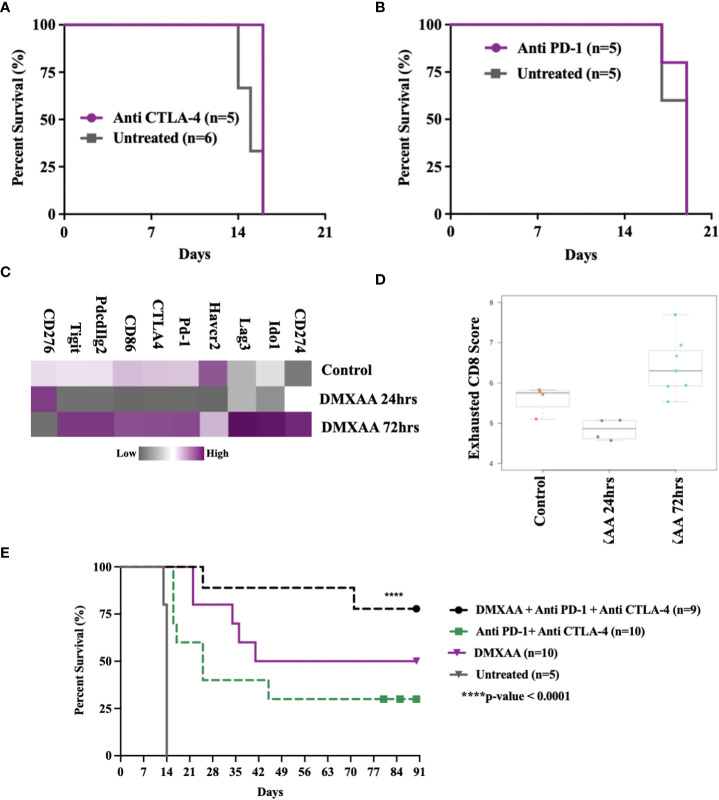
Therapeutic synergy of STING activation and immune checkpoint blockade in murine UPS tumors. **(A, B)** 100,000 UPS + mCherry and luciferase cells were injected intramuscularly on day 0. **(A)** 250 μg mouse anti-CTLA4 monoclonal antibody or **(B)** 250 μg mouse anti-PD1 monoclonal antibody were injected intraperitoneally, on days 7, 10, and 13 following UPS engraftment. **(C)** NanoString mRNA expression profile of common immune checkpoint markers. Upregulated expression is shown in purple and downregulated expression is shown in grey. Control UPS tumors (n=4), 24 hours post DMXAA UPS tumors (n=4), 72 hours post DMXAA UPS tumors (n=7). **(D)** Exhausted CD8 pathway score using Nanostring Technologies.**(E)** Anti-PD-1 (250 μg) + anti-CTLA4 (250 μg) were injected intraperitoneally on days 9, 11, 15, 18, 22, 25, 29, and 32 following UPS injection (black and green), DMXAA (18 mg/kg) was injected i.t. on day 7 (purple).

This murine model of UPS is resistant to anti-CTLA4 and anti-PD-1 monotherapy (**Figures 8A, B**) and documented by others (29). We have observed late UPS tumor relapses in mice treated with DMXAA after near complete tumor eradication (relapses, **Figures 1C, D**). As we have also observed increased CD8+ T cell infiltration and cytotoxic lymphocyte scores following STING treatment of UPS tumors, we sought to determine genes associated with negative immune regulation were upregulated in UPS tumors after i.t. DMXAA. We observed upregulation of *Ido1*, *Lag3*, *Pd-1*, *Ctla4*, *Pdcd1lg2*, and *Tigit* transcripts 72hrs post DMXAA treatment compared to control UPS tumors (**Figure 8C**). Mean exhausted CD8 scores were also at this timepoint (**Figure 8D**), collectively implying an opportunity to increase therapeutic outcomes in STING treated UPS tumors by the addition of immune checkpoint inhibition (ICI) therapy.

The additional of both anti-PD1 and anti-CTLA4 therapy improved STING-mediated tumor clearance from 50% to 80%. We also observed 30% tumor clearance using combination ICI therapy without STING therapy in this UPS model (**Figure 8E**). These results suggest (i) there is baseline negative immune checkpoint regulation in this model that can be therapeutically targeted using combination ICI therapy, but not monotherapy and (ii) STING activation results in further upregulation of negative T cell co-stimulatory pathways that can be targeted to improve tumor clearance.

## Discussion

Soft tissue sarcomas (STS) are rare, high-fatality cancers that are poorly responsive to systemic therapies (6, 41–44). Recent clinical trials have persistently failed to show significant clinical benefit for patients with advanced STS treated with immune checkpoint inhibitors (6, 12, 45), and other immune-based therapies (7, 9, 46–50). While considerable heterogeneity exists within the complex karyotypes of STS, the TIME of most STS is immunologically cold, which predicts poor sensitivity to immune therapies (22). Using a transplantable, immune competent, orthotopic murine model of UPS that recapitulates the lymphocyte poor TIME of most STS, we sought to determine if STING immunotherapy could dismantle the immunosuppressive features of this model and promote immunogenic tumor eradication. Here, we demonstrate that i.t. STING activation can promote tumor necrosis, lymphocyte mediated tumor clearance and durable tumor eradication in up to 60% of UPS-bearing mice following a single injection of a small molecule STING agonist. Additionally, i.t. STING therapy was also effective on systemic sites of disease in the lung, and in mice that cleared tumor following therapy, durable immunity against UPS re-challenge was present.

While there have been numerous studies examining the therapeutic potential of STING agonism in solid tumor models (19, 28, 51–53), this is the first detailed examination of STING therapy in an immunologically cold model of sarcoma. Recently, Wolf et al., did test a STING agonist, ADU-S100, in combination with an IL-2 superkine (H9-MSA) using the methylcholanthrene carcinogen model of sarcoma (51). This model of UPS has a high mutational burden (2000 non-synonymous mutations/tumor) compared to the KP UPS model (18 non-synonymous mutations/tumor) and is more representative of the mutational burden observed in human cancers that are sensitive to immunotherapies (54, 55). Conversely, the TIME of the KP model of UPS contains a paucity of lymphocytes, is enriched in CD206 immunosuppressive macrophages, and is resistant to immune checkpoint blockade (29, 56), thus recapitulating the immunotherapy resistant phenotype common to most sarcomas. We do recognize that our UPS model used here is driven by Kras and p53 mutations, which are also used to induce lung and pancreatic carcinomas in mice. Indeed, there is also evidence that STING activation can induce therapeutic responses in these models (37, 57), suggesting that these mutations or associated downstream effector pathways may support sensitivity to STING therapy.

Similar to other studies, we have shown that STING-mediated clearance of tumor cells in this UPS model is dependent on functional lymphocytes (28, 52, 53). Additionally, our data importantly shows the resultant systemic treatment effect following i.t. STING activation as we observed durable survival in mice with synchronous extremity and lung tumors following treatment of the extremity tumor. This, coupled to the rejection of UPS re-challenge in the leg or lung highlights the persistent anti-sarcoma systemic adaptive immunity following a single treatment of STING activation. This is clinically important as the lung is the principal visceral site of STS metastases or systemic relapse (58, 59). These data justify further study into how STING-based immunotherapy for primary sarcomas could be used to systemically eradicate micro-metastases or prevent relapses following local control procedures.

A central process of STING immunotherapy is the induced cooperation of rapid innate immune responses with persistent adaptive immune-based elimination of cancer cells. Numerous studies have demonstrated impaired STING signaling or downregulation of STING in cancer cells, suggesting the stromal constituents of the TIME are the critical targets of STING activation (60–63). As macrophages are highly sensitive to STING agonists (64–66) and are abundant in both human and pre-clinical sarcoma models (29, 39, 56, 67–69), we hypothesized that a reduction in tumor associated macrophages (TAMs) would mitigate the therapeutic response to STING agonism. Monocytes are known to contribute to the TAM populations, and the CCR2/CCL2 signaling is critical for TAM recruitment from monocyte lineages (55). We did observe a 75% reduction in UPS TAMs in the CCR2 KO line but did not observe any change in long-term survival and instead observed earlier onset of tumor volume and BLI reductions following treatment. It is possible TAMs are not the dominant effector cell of small molecule STING agonists in this model and STING signaling occurs *via* other cell populations such as tumor resident DCs (21), endothelial cells (19) or remaining macrophage pools. Alternatively, inhibition of the CCL2/CCR2 axis is associated a decrease in CD206 immunosuppressive macrophage populations (38) and thus a reduction in CD206 TAMs in CCR2 KO mice may provide a more inflamed and sensitive environment for STING responsiveness.

An interesting observation in these experiments were the late tumor relapses following STING therapy. In these mice (30%), tumors substantially regressed and were not palpable, but quickly rebounded 2-3 weeks after treatment. Transcriptomic data of STING treated tumors did show increased expression of markers associated with T-cell inhibition and T-cell exhaustion which could explain late treatment resistance. Supporting this, we observed improved tumor clearance from 50% to 80% when STING therapy was combined with immune checkpoint inhibition (anti-CTLA4 and anti-PD1). These observations are consistent with pre-clinical studies in other cancer models showing STING-dependent upregulation of negative immune checkpoints and improved therapeutic responses when STING agonism is combined with immune checkpoint blockade (37, 52, 70). As there is considerable clinical enthusiasm to understand which clinical STS will benefit from immune checkpoint blockade, the addition of intra-tumoral STING therapy may provide an opportunity to improve response rates across more STS subtypes.

We acknowledge that there are limitations within the present study. Firstly, it has been well characterized that DMXAA is murine-specific STING agonist and does not activate human STING (71). We elected to use DMXAA in this study as proof of concept given the documented efficacy, known toxicities and well-defined dosing parameters of this small molecule STING agonist. Over the past decade, numerous small molecule STING agonists capable of activating human STING have been developed (51, 52, 72) and while some of these agents are now being tested in clinical trials, there remains much to learn regarding how these new agents should be administered and dosed locally, systemically, and in concert with other therapies. Future studies are ongoing evaluating these new agents in different genetic models of STS. Another limitation pertains to the cell-line derived UPS tumors used in this study. We and others have shown that engrafted KP UPS tumors demonstrate increased spontaneous lymphocytic infiltrated compared to spontaneous KP UPS tumors (56). Therefore, these engrafted tumors may be more sensitive to STING therapy and future studies evaluating spontaneous tumors will be required. Engraftable tumors enabled a more consistent, reproducible, and feasible experiments as we could predictably induce tumors and begin therapy using consistent timelines. Further work will be completed to delineate the tumor antigens involved in this UPS model following STING activation.

## Conclusion

To our knowledge, this is the first study to evaluate STING immunotherapy in the KP model of UPS. Like most human STS, the KP sarcoma model has an immune-suppressed TIME and is resistant to immune checkpoint blockade. We have shown that a single treatment of intra-tumoral STING activation can induce immune-mediated sarcoma clearance locally and systemically. These results justify further study into the clinical translation of STING immunotherapy for sarcomas.

## Data availability statement

The original contributions presented in the study are included in the article/**Supplementary Materials**. Further inquiries can be directed to the corresponding authors.

## Ethics statement

The animal study was reviewed and approved by University of Calgary Health Sciences Animal Care Committee (#AC19-0072).

## Author contributions

KLM contributed to project conception, completion, study design, and produced the first draft of the manuscript. KMH and KNH contributed to experiment completion and final manuscript production. AKS contributed to study design and experiment execution. FJZ, DJM, and FRJ contributed to study conception and project design. MJM contributed to project conception, design, manuscript development, and funding acquisition. All authors contributed to the article and approved the submitted version.
