# Supplementary material for: Intratumoral STING activation causes durable immunogenic tumor eradication in the KP soft tissue sarcoma model
Source: Front Immunol. 2023 Jan 9;13:1087991. doi: 10.3389/fimmu.2022.1087991 (PMC9868147; doi:10.3389/fimmu.2022.1087991)
Supplement: Supplementary file 1 [file DataSheet_1.docx]

Supplementary Material

**Figure S1.** **Sex differences in overall survival to DMXAA treatment.** 100,000 UPS + mCherry and luciferase cells were injected into C57Bl/6 mice intramuscularly on day 0. C57Bl/6 mice were treated with either a single, double, or triple dose of DMXAA. The single dose DMXAA mice received either 18 mg/kg or 25 mg/kg of DMXAA 7 days after UPS cell line injection. The double and triple dose DMXAA mice received 18 mg/kg of DMXAA 7 and 14 days or 7, 11, and 14 days after UPS cell line injection respectively. The female DMXAA treated mice are shown in green and the male DMXAA treated mice are shown in black.
