# Supplementary figures and images for: Intratumoral STING activation causes durable immunogenic tumor eradication in the KP soft tissue sarcoma model
Source: Front Immunol. 2023 Jan 9;13:1087991. doi: 10.3389/fimmu.2022.1087991 (PMC9868147; doi:10.3389/fimmu.2022.1087991)

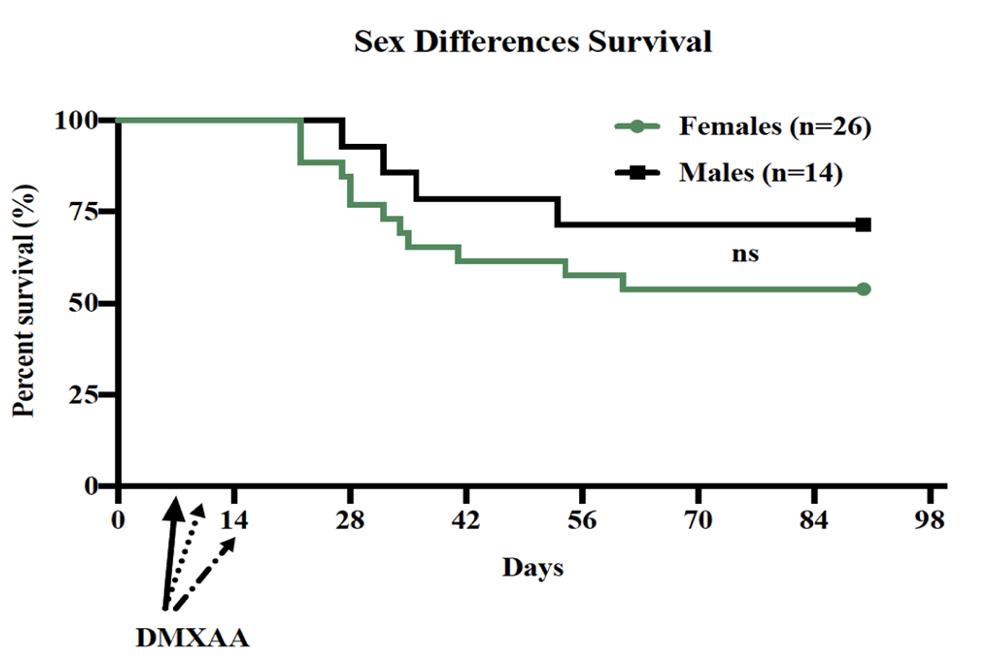

Supplement: Supplementary file 2 [file Image_1.tiff]
